# 309. Multidrug-Resistant Organism Colonisation and Surgical Site Infections Following Liver Transplantation

**DOI:** 10.1093/ofid/ofaf695.105

**Published:** 2026-01-11

**Authors:** Mohamed Nasra, Nirbaanjot Walia, Katrina Tan, Marie Sinclair, Avik Majumdar, Jason Trubiano, Graham Starkey, Marcos Perini, Jason Kwong, Adam Testro, Olivia Smibert

**Affiliations:** Austin Health, Melbourne, Victoria, Australia; Austin Health, Melbourne, Victoria, Australia; Austin Health, Melbourne, Victoria, Australia; Austin Health, Melbourne, Victoria, Australia; Austin Health, Melbourne, Victoria, Australia; Austin Health, Melbourne, Victoria, Australia; Austin Health, Melbourne, Victoria, Australia; Austin Health, Melbourne, Victoria, Australia; Austin Health, Melbourne, Victoria, Australia; Austin Health, Melbourne, Victoria, Australia; Austin Health, Melbourne, Victoria, Australia

## Abstract

**Background:**

Liver transplant (LTx) recipients are at increased risk of surgical site infections (SSIs). We explored potential risk factors for SSIs post-LTx including colonisation with multidrug-resistant organisms (MDRO) and aimed to assess the impact of perioperative antibiotics targeted at colonising MDRO on the subsequent risk of SSI.

**Methods:**

A retrospective analysis of adults undergoing LTx at a tertiary referral centre between 2015–2022 was conducted. MDRO colonisation status was determined by routine rectal screening for MDRO including vancomycin-resistant enterococci and extended-spectrum beta-lactamase-producing (ESBL) and carbapenemase-producing Enterobacterales (CPE) at time of LTx. The primary outcome was SSI defined using CDC/NHSN criteria. Clinical features related to the patient and donor organ were retrieved from medical records. A multivariable analysis was conducted to evaluate the impact of MDRO colonisation and targeted antibiotics on 30-day SSI risk.

**Results:**

Of 462 patients who underwent LTx, 157/462 (34.0%) were colonised with MDRO (131 [28.4%] VRE and 38 [8.4%] ESBL/AmpC/CPE). The 30-day SSI rate was 11.9% (55/462), including 16.4% superficial, 5.2% deep incisional and 78.4% organ-space infections. Post-operative biliary complications (aOR 2.1 95% CI 0.98-4.27; P=0.048) and post-LTx haemodialysis (aOR 3.84; 95% CI 1.75-8.17, P= 6e-04) were significantly associated with increased SSI risk on multivariable analysis. There was no association between MDRO colonisation and risk of SSI following LTx (aOR 1.47; 95% CI 0.79-2.71, P=0.2195)). Among MDRO colonised patients (n=157), the use of perioperative antibiotics targeted at colonising MDRO was not associated with reduced SSI risk (aOR 0.40; 95% CI 0.08-1.4; P=0.189).

**Conclusion:**

SSIs following LTx most commonly involve deep organ spaces. MDRO colonisation was not independently associated with an increased SSI risk, and receiving directed-perioperative antibiotic prophylaxis did not decrease SSI risk.

**Disclosures:**

All Authors: No reported disclosures

